# Effect of pasture and intensive feeding systems on the carcass and meat quality of buffalo

**DOI:** 10.5713/ab.21.0141

**Published:** 2021-06-23

**Authors:** Michela Contò, Giulia Francesca Cifuni, Miriam Iacurto, Sebastiana Failla

**Affiliations:** 1 Consiglio per la Ricerca in Agricoltura e l‘Analisi dell’Economia Agraria (CREA), Centro di ricerca Zootecnia e Acquacoltura (Research Centre for Animal Production and Aquaculture), Via Salaria 31, Monterotondo, Rome 00016, Italy

**Keywords:** Buffalo, Feeding System, Grazing, Meat Quality

## Abstract

**Objective:**

This work was carried out to evaluate the effect of pasture (PA) feeding on buffalo meat quality compared with buffaloes reared intensively with the use of corn silage as a forage base or alternatively with polyphite meadow hay (PH).

**Methods:**

Thirty Mediterranean bull buffaloes were distributed into three experimental diet groups: maize silage (MS), PH, and PA. The animals were slaughtered at a live weight of 250 kg, and carcass and meat quality were evaluated. After 7 days of ageing, physical and chemical parameters of *longissimus thoracis* muscle were determined. To evaluate lipid oxidation the thiobarbituric acid reactive substances was tested at 7 and 14 days, and also the fatty acid profile was recorded by gas chromatography.

**Results:**

The PA group, even if it showed carcass parameters lower than those of the silage maize group, reported a good meat percentage (60.59% vs 58.46%, respectively) and lower fat percentage (p<0.001). PA-fed animals showed meat redness, and even if only on raw meat, shear force was higher than the others. Low values of conjugate linoleic acid, polyunsaturated fatty acids, and n-3 were reported in the silage maize group. Principal component analysis (PCA) clearly showed the influence of different diets on meat quality, and PCA1 and PCA2 explained 82% of the variability.

**Conclusion:**

Buffaloes reared on PA had meat with high nutritional value even if they showed poor carcass performance compared to the animals fed on MS. Buffaloes fed on polyphite hay were in an intermediate position, similar to grazing animals, according to the same nutritional determinations.

## INTRODUCTION

Italy, among Western countries, has more buffalo heads; in 2019, the buffalo population was estimated to be 402,290 heads. The principal Italian buffalo product is “Mozzarella di Bufala DOP”, but in the last decade, buffalo meat has also increased by 16% (FAOStat data 2019). This trend is due to high meat nutritional values, such as low cholesterol levels, high iron content, and low fat content é [1–4] and because buffalo meat is comparable to beef in many of its physicochemical, nutritional and functional properties and sensory attributes [5,6].

Buffaloes are usually bred in South Italy in intensive systems for mozzarella cheese production [7], where, except for the summer season, there are pastures (PA) that could be used for semi-extensive breeding of buffalo males, leading to increased producer income thanks to lower production costs due to the rusticity of the species, which shows good digestive capacity for roughage [8].

In order to meet market demands and breed high-quality standard animals, several studies showed the influence of feeding on carcass quality and meat characteristics [8]. More recently, it has been demonstrated that using adequate feeding strategies for fattening buffaloes favourable nutritional characteristics can be achieved [8].

Studies to improve the performance and carcass quality of buffaloes [9] noticed that the supplementation of PA with concentrate enhances the growth and carcass characteristics, whereas animals grazing only PA had a more favourable fatty acid profile.

Previous our research [1] has shown that the type of finishing diet (hay or maize silage [MS]) for fattening buffaloes has a significant effect on carcass characteristics and meat quality. Finishing on MS produces carcasses with higher fat deposition and meat with a less favourable fatty acid profile (i.e. a lower P/S ratio and α-linolenic fatty acid content) in relation to human health. Moreover, PA-raised animals are lower in overall intramuscular fat, and it would seem that grass-fed animals could have lower cholesterol content and higher concentrations of antioxidant molecules, precursors of vitamins, minerals, n-3 fatty acids and increased trans vaccenic acid, used for the de novo synthesis of conjugate linoleic acid (CLA) [10]. Also, the meat of grass-fed beef cows could be darker than that of cows fed on concentrate [11].

Consumers’ interest is focused on environmental sustainability, animal welfare and the nutritional values of animal products, and though this order changes according to the level of education, lifestyle choices and customs with a marked temporal effect, consumer behaviour is generally linked to healthy choices [12]. In particular, for meat products, the system of raising can inform these interests, while also improving the nutritional value of meat; indeed, forage or grass-feeding can modify the fatty acid composition and antioxidant content to improve nutritional, organoleptic and colour stability [13,14].

The aim of this work was to evaluate the effects of PA feeding on the carcass composition and physical-chemical and nutritional qualities of buffalo meat, in comparison with buffaloes reared intensively with the use of corn silage as a forage base or alternatively with polyphite meadow hay.

## MATERIALS AND METHODS

### Animals and diets

The experiment was carried out at the Research Centre for Animal Production and Aquaculture (CREA-ZA) in Central Italy, and the experiment was conducted for approximately 6 months from January to June. Thirty Italian Mediterranean buffalo bulls, after weaning (approximately 3 months), were reared together until 5 months of age on multiple boxes. After that, they were randomly distributed into three experimental diet groups: 10 animals were fed on MS *ad libitum*; 10 animals were fed on polyphite meadow hay (PH) *ad libitum*; and 10 animals were fed on PA. All animals received a supplement of maize grain (0.8 kg/d per 100 kg live weight) and protein concentrate (500 g head), and they had free access to water. The MS and PH groups were kept in a box with free access to a paddock, while the PA group was reared in grazed meadows with *Gramineae* as the principal component.

To determine the chemical composition of the diets (Table 1), a sample of MS and hay was taken monthly for the experimental period, while the PA sample was taken monthly from three boxes to prevent access by the animals, as reported by Fruet et al [15]. A single sample for maize grain and protein concentrate was taken for the total experimental period.

Each sample of MA, hay, and PA was combined to form a single sample that was suitably mixed. Maize grain and protein concentrate were dried in a forced-air oven at 65°C for 72 hours and then ground to a mesh size of 1 mm. Analysis of the dry matter (DM), crude protein, and ether extract (EE) from the diets was conducted according to the AOAC [16]. The concentrations of neutral detergent fibre and acid detergent fibre were analysed according to the method described by Van Soest et al [17]. The principal fatty acids were determined with the same method reported later for meat.

### Carcass parameters and physical analyses

At approximately 250 kg of live weight the animals were slaughtered by captive bolt pistol and exsanguination in experimental slaughterhouses. After slaughter and removal of the head, skin and feet, the digestive tract and rumen were removed and weighed before and after emptying. Carcasses were split into two sides and chilled at 2°C±1°C; after 7 days of ageing, the carcass sides were evaluated through visual assessment by a trained and experienced evaluator for conformation and fatness according to the EU standard method SEUROP classification [18]. Each class of both scales (conformation and fatness) was subdivided into 3 subclasses, obtaining 18 subclasses for conformation and 15 for fatness evaluation.

**Figure 1:**
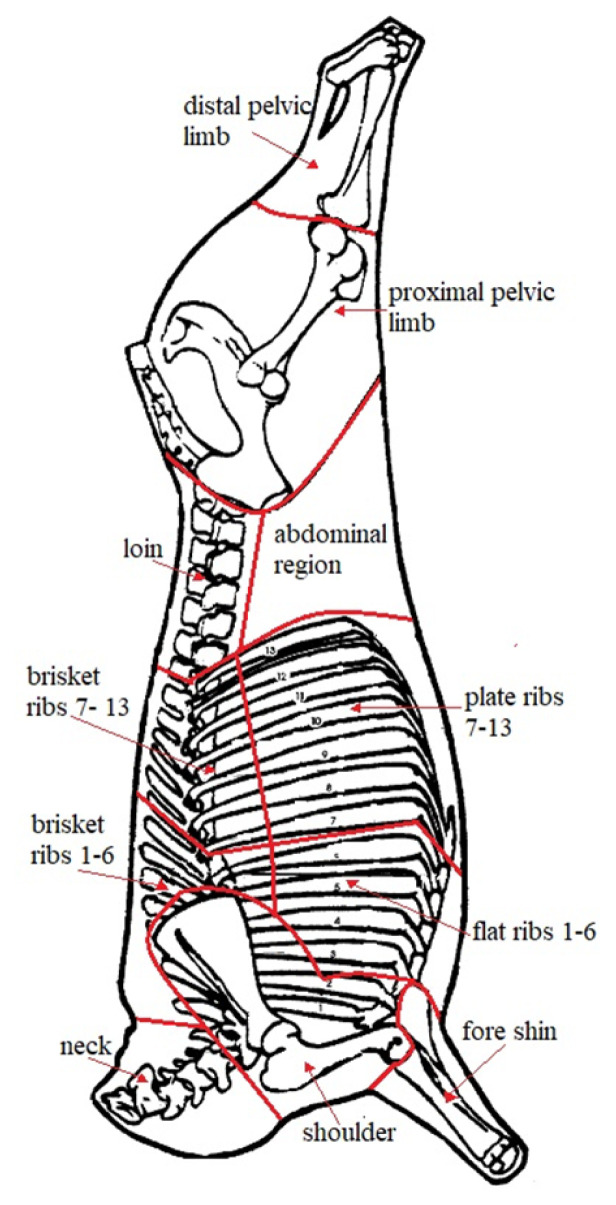
Carcass subdivision into 11 anatomical regions, from the right half carcass, neck, fore shin, shoulder, brisket 1–6, brisket 7–13, flat ribs 1–6, plate ribs 7–13, loin, distal pelvic limb, proximal pelvic limb and abdominal region.

From the right half carcass, 11 anatomical regions were dissected, 7 from the hindquarter (neck, fore shin, shoulder, brisket 1–6, brisket 7–13, flat ribs 1–6, plate ribs 7–13) and 4 from the forequarter (loin, distal pelvic limb, proximal pelvic limb and abdominal region), as reported in Figure 1. Carcasses and all regions considered were weighed and dissected for meat bones and fat percentage determination.

After 7 days of ageing, the *longissimus thoracis* muscle (LT), between 7th to 11th thoracic ribs, was taken from each half carcass and divided into two portions. The portions were, also, divided in three 2.5 cm steak slices and two of them used to perform physical and chemical analysis at 7 days, and the other portion was stored in a polyethylene bag under vacuum at 4°C until 14 days of ageing to determine oxidative processes. The chemicals analyses were performed in duplicate and the means were used for statistical data analysis.

Muscle pH was measured with a portable Hanna pH metre with an Inlab 427 probe, with temperature compensation performed by making a scalpel incision in the muscle, and water-holding capacity (WHC), measured as water loss in raw and cooked meat (CL), was determined according to Honikel [19]. Cooked samples were obtained cooked a slices in a plastic bag in water bath at 80°C until a 75°C at core and cooled for 45 min in running water. The shear force on raw and cooked meat was evaluated using an Instron 5543 equipped with a Warner–Bratzler shear force device (WBS) as described in detail by Christensen et al [20], applied on six sample blocks 2×1×1 cm cross section and cut perpendicularly of the fibres direction. For cooked samples the same procedure described above was used and the means were used to statical analysis.

Meat colour was evaluated using the CIELAB system to estimate L*, a*, and b* (lightness, redness, and yellowness, respectively) and calculate ΔE* with a Minolta CM-2006, as reported in detail by Ripoll et al [21]. For each sample six determination was performed and the means were used for statistical data analysis.

### Chemical analyses

Chemical composition, DM, ash, EE and protein of the diets and meat at 7 days of ageing were determined by AOAC [16].

Total and insoluble collagen content was determined according to Christensen et al [20] by quantification of hydroxyproline. For total collagen 5 g of chopped meat samples were hydrolyzed in 30 mL of 6 M HCl at 110°C overnight; instead for insoluble collagen, before hydrolyzing process, samples were cooked for 2 h at 90°C in 20 mL of 0.9% NaCl solution, after cooled samples were centrifuge and the suspension in aqueous phase was collected by filtrating and add to the pellet. Pellet and filter was hydrolyzed as reported above. After hydrolyzing process samples were neutralized at 7 pH, an aliquot was incubated at room temperature for 20 min with the oxidative solution of Chloramina-T, later the same aliquot was incubated at 60°C per 15 min with colorimetric solution, after cooled in ice bath, samples were read at 560 nm. Total and insoluble collagen were calculated by standard curve of hydroxyproline standard for conversion factor 8.

Lipid oxidation was analysing at 7 and 14 days after slaughter by thiobarbituric acid reactive substances assay (TBARS), that measuring the level of malondialdehyde (MDA), a principal lipid oxidation product, following the procedure reported by Bergamo et al [22]. Meat samples were homogenized in water, and after proteins were precipitated with trichloroacetic acid (TCA 10%), the deproteinized sample was incubated with thiobarbituric acid at 90°C for 30 min and subsequently detected by HPLC. An aliquot, 20 μL, of sample was inject into C18 reverse phase column (4.6×250 mm 5 μm) with isocratic mobile phase of Buffer phosphate 5 mM pH 7:Acetonitril 85:15 (v:v) and read by fluorescence, λEX = 515 nm; Λem = 543 nm. The MDA peak was identified by the elution profile of authentic standard. A calibration curve was performed using 1,1,3,3-tetraethoxypropane (TEP) solutions of concentrations varying from 0.007 to 1.25 mg/mL. The TBARS concentration was expressed as mg of MDA/kg of meat and triplicate analyses were performed for all samples. The limit of detection (LOD) and limit of quantitation (LOQ) were found to be 0.00042 and 0.0014 mg/mL, respectively.

Fatty acids were extracted by chloroform:methanol (2:1 v/v), before to perform the fats extraction C19:0 fatty acid was added as to internal standard on samples. Fat was methylated with methanolic KOH, and methyl esters were injected in a gas chromatography flame ionization detector with a fused silica capillary column coated with 100% cyanopropyl polysiloxane as described in detail in Cifuni et al [1]. Peak identification was performed by comparing the sample peaks with Supelco37 (Merck-Sigma Aldrich, Bellefonte, PA, USA) standard peaks, expressed as the percentage of total fatty acid methyl ester. The amounts of saturated fatty acids (SFA), monounsaturated fatty acids (MUFA), polyunsaturated fatty acids (PUFA), n-6/n-3 PUFA ratio, PUFA/SFA ratio, thrombogenic index (TI) and atherogenic index as reported in Ulbricht et al [23] were calculated.

### Statistical analysis

All data were subjected to analysis of variance with a mono factorial model, using procedure general linear model of SAS (SAS Inst. Inc., Cary, NC, USA), and the level of significance between the groups was determined according to Tukey’s test using p<0.05 as the limit to identify significant differences. In addition, principal component analysis (PCA) was performed by SAS to identify the components that absorb greater variability and was able to separate the three feeding systems. For this analysis, only the qualitative parameters of meat that showed significant differences between experimental groups were used.

## RESULTS

### Slaughter performance

The PA group reached the final weight at the highest slaughter age (+48 days compared to the mean of the other groups p<0.001) and consequently a lower (p = 0.015) daily gain (ADG), also showing the highest gastro enteric content (43.0 vs 21.6 kg of the MS group), with consequent minor net live weight; however, the carcass weight of these animals was not significantly different from the PH group, although the weight was −28 kg lower than the MS group (Table 2).

Animals fed MS significantly differed from the PA group in dressing percentage (56.9% vs 49.5% p<0.001). The conformation score was similar for MS and PH, both differing by PA (p<0.001), while the PA and PH groups showed the leanest carcasses according to the fatness score. This result was comparable to the carcass tissue percentage, where MS carcasses showed a fat percentage greater than +5%; conversely, the PH and PA groups had significantly higher meat percentages (62.09% and 60.61%, respectively, compared to 58.46% of the MS group). For bone percentage, the MS and PA showed opposite trends, with PH in the middle position.

### Anatomical regions

The percentage of anatomical regions (Table 3) showed a few significant differences. The proximate pelvic limb (27.7% on the average) and shoulder (12.60% on the average), on the hindquarter, were the most representative regions of the carcass. The regions where subcutaneous fat was usually stored had a higher incidence in MS carcasses, as did the abdominal region (4.03% vs 3.72% means of PH and PA), while the distal pelvic limb reported the opposite trend (p<0.001). The other anatomical regions did not show significant differences.

### Physical and chemical quality

Regarding physical analysis (Table 4), pH was higher for the PA group (p<0.001), while MS showed a lower value (5.73 vs 5.54, respectively). No difference was found in WHC %; instead, cooking loss reported a higher value for the MS group than for the PH group (p<0.001).

Buffalo meat from the silage maize diet showed the highest L* value and the lowest a* value compared with the other groups (p<0.001 for both), while the yellowness index was highest in the MS group (14.53). The other two groups did not show significant differences from each other.

WBS on raw meat reported a lower value in the MS group than the others (p = 0.013), and no difference was found in WBS on cooked meat.

Buffaloes fed MS and polyphite hay showed a higher fat percentage (p<0.001), while no significant differences were reported for the other proximate compounds.

Total collagen was higher in the PA and PH groups than in the animals fed MS (p = 0.004), while insoluble collagen was not significant.

The oxidation parameter, TBARS, was higher in the MS group than in the PA group, 0.16 vs 0.11 mg MDA/kg at 7 days. As we expected, TBARS increased from 7 to 14 days of storage time, particularly in the MS group, which had the highest value (p<0.001).

The fatty acid profile (Table 5) showed the effects of different diets. For SFAs, our data reported significant differences only for myristic acid (C14:0) and palmitic acid (C16:0), with the highest value for the MS group (p<0.05 for both fatty acids), while for MUFAs, the C16:1 and C18:1 cis-11 were lower in the same group. The greatest differences were found for some PUFAs and n-3 fatty acids; in fact, PA feed increased the intake of polyunsaturated n-3 fatty acids (Table 1).

In particular, the PA group reported a higher value than the MS group in CLAcis-9 trans-11 (0.35% vs 0.22%), long chain PUFAs (LC PUFA n-3) such as eicosapentaenoic acid (C20:5 n-3), docosapentaenoic acid (C22:5 n-3), and docosahexaenoic acid (C22:6 n-3) and consequently showed the lowest n-6/n-3 ratio and atherogenic and TI (p = 0.003 and p = 0.006, respectively).

**Figure 2:**
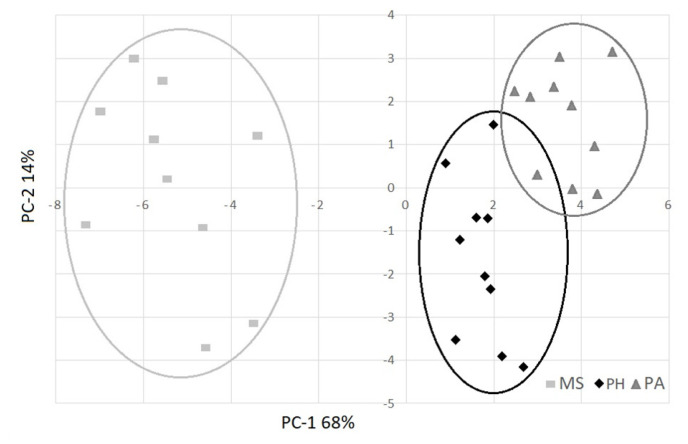
Scores on two principal components for meat samples produced from buffaloes fed with maize silage (MS), polyphite meadow hay (PH) and pasture (PA).

**Figure 3:**
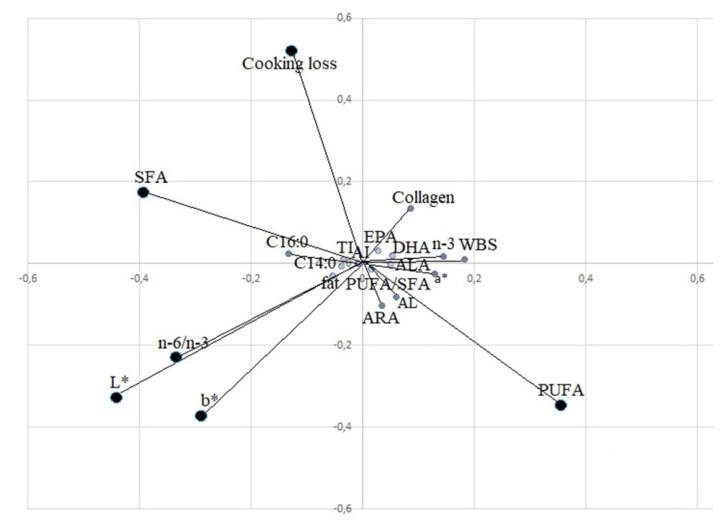
Principal component analysis (PCA) loading plot of the meat variables that absorbed the major variability on the first two PCs. MUFA, mono unsaturated fatty acid; SFA, saturated fatty acids; PUFA, poly unsaturated fatty acid, ARA, arachidonic acid; ALA, α-linolenic acid; AL, linoleic acid; EPA, eicosapentaenoic acid; DHA, docosahexaenoic acid; WBS, warner bratzel shear force; AI, atherogenic index; TI, thrombogenic index.

PCA was used to identify a classification criterion for meat samples, using the feeding system as a grouping variable. PCA1 and PCA2 explained 82% of the variability and clearly separated the three groups (Figure 2). Using only the significant qualitative parameters indicated in the tables, 0.89 R^2^ and 0.21 RMSE were obtained. The loading plot (Figure 3) clearly showed the importance of fatty acids to determine the qualitative differences of meat for SFA, PUFA fatty acids and n-6/n-3 ratio. For physical parameters, colour (lightness and yellowness) and cooking loss were important to separate the three groups. The PA group was in opposition to the n-6/n-3 ratio, SFA and colour parameters (L* and b*) because it showed redder and leaner meat, while the MS group, which showed lighter and fatter meat, was in the opposite position with respect to PA, where SFA, L*, and b* absorb major variability. The PH group was in the intermediate position, characterized by PUFAs, in the opposite position of cooking loss.

## DISCUSSION

Feeding systems influenced productive performance and meat quality, as reported by several authors reviewed by Muir et al [24]. As we expected, major differences were found between the buffaloes fed silage maize and the PA group, which were the two opposite livestock management practices, while the animals fed polyphite meadow hay were in the intermediate position.

Grazing caused lower ADG than the other groups due to both energy consumption for major activities linked to grazing and a ration richer in fibre; this last factor also affects the content of the gastroenteric apparatus and therefore the carcass yield with about −28 kg for carcass weight than buffalo fed MS, despite slaughter at a similar live weight, which consequently affects the carcass yield [9,25]. However, Lambertz et al [9] found a difference of 200 g in ADG in animals bred with only PA feeding relative to those fed in pens; this more marked difference was probably due to the absence of concentrate integration in grazing animals. As reported by Lapitan et al [7], the effect of poorer feeding on ADG was less distinct in buffalo than in beef due to its greater ability to utilize roughage. The conformation and adiposity scores among the intensively reared animals (MS and PH) showed the same trend reported by Cifuni et al [1] in animals older than 16 months.

Buffaloes fed roughage or by grazing showed significantly leaner carcasses [1,8] for both cattle and buffalo, confirming that diet influenced fat deposition.

Anatomical regions expressed in kg followed the carcass weight trend, with the higher weight in MS carcasses for all anatomical regions considered, reaching a difference of 9.5 kg compared to animals raised on PA. The anatomical regions included in the percentage of total carcass weight (Table 3) were similar to those reported by Lapitan et al [7] and Lambertz et al [9], which showed a significantly lower incidence in briskets between animals fed on only PA compared to those who received integrated feeding with concentrate. MS feeding significantly increased the incidence of the abdominal region, the area of choice for fat deposition, because the animals that received high energy intake, even if young, were fattened.

Meat quality (Table 4) was significantly affected by the feeding system. High pH in meat from animals fed on PA was reported by several authors [10,13]. Animals fed a richer diet generally accumulate more glycogen in muscles, which, after slaughter, due to the transformation of glycogen into lactic acid, contributes to a decrease in pH during ageing [11]. Furthermore, a greater adipose panicle in the carcass determines lower heat exchange during ageing, which can cause a slower decrease in temperature, affecting the pH [6]. Additionally, the difference in cooking loss could be due to the higher fat content in the MS group that was partly lost in the cooking liquid; in fact, meat containing a high percentage of fat results in greater cooking loss than lean meat [1].

The MS group was lighter than the others, in fact showed a higher L* colour values than the other groups (p<0.001), probably due to the greater amount of fat and to lower pH, factors that can be considered a contributing cause of the same phenomena; in fact, the high incidence of subcutaneous fat causes a slower cooling rate of carcasses, corresponding to a faster pH decrease [11] [4,21].

Colour is an important quality parameter that guides the consumer’s choice, and if the ΔE* value exceeds the threshold of just noticeable difference (JND = 2.3), it means that differences in colour are perceived by the human eye (CIE, 1976). The MS group indeed showed evident differences between animals fed on hay (ΔE* = 5.3) and grazing (ΔE* = 8.7); between these two groups, the grazing animals tended to show a darker colour, although not significantly, with non-perceptible colour differences (ΔE* = 1.4). In contrast, Huuskonen et al [26] did not detect significant differences in the meat colour of cattle finished on PA compared with those finished on MS [24], probably because no differences in fat score were reported. Furthermore, Marrone et al [8] found in meat from buffaloes fattening with rye grass higher values of a*, b*, and lower lightness compared to concentrate fattening group.

Tenderness was considered by consumers to be the most important component of meat quality. In cooked meat, the diets did not affect the WBS parameter, as reported by several authors [1,8,9], but probably due to fat, the lowest value of WBS in raw meat was reported in the MS group (p = 0.0134), and a negative correlation between fat and shear force, even if limited, was highlighted by Fiem et al [27], who also described a negative correlation between shear force and lightness. Similar data between concentrate-fed and grass-fed animals were reported by Nuernberg et al [13] in beef. Even the greater presence of total collagen could influence the WBS value [20]; in fact, animals with a lower quantity of collagen (MS group) had a meat that was tender, and the differences were erased considering the percentage of insolubility, which partly explains the lack of a significant difference between groups in WBS on cooked meat [20]. Some authors associated the increase in collagen with lower daily weight gain, low protein turnover and constant movement of animals during grazing [28]. The proximate composition of meat once again underlines the limited accumulation of fat in grazing animals [9,10,25].

Lipid oxidation showed the expected trend; in fact, buffaloes fed by grazing showed a lower TBARS than MS, but the difference was more considerable at 14 days of ageing, as reported by Nuernberg et al [13] at 5 and 10 days for cattle fed on grass or concentrate. Most likely, the animals on PA retained more vitamin E, polyphenols and carotenoids with a high antioxidant effect [13,29]. Lipid oxidation increased with ageing time because of a normal degradation process [30], but even if buffalo meat had a high iron concentration [4], the oxidation process was not particularly high compared to the value reported in bovines by Nuernberg et al [13].

Several authors considered the importance of grass feeding to improve the CLA, n-3 and n-6 fatty acids [10,13,14, 25,26]. CLA cis-9 trans-11 has important nutraceutical effects on human health. A switch from a concentrate-based diet to PA has been shown to increase CLA content [13,14]; in fact, CLA cis-9 trans-11 in our data was higher in PA than MS (p<0.001). Grazing and green hay improved the trans vaccenic acid content, an important fatty acid for de novo synthesis of CLA, as reported by Daley et al [14], who also underlined in his review that the rumen in animals fed on PA or green forage during ruminal fermentation shows an optimal pH value for growing *Butyrivibrio fibrisolvents*, an important rumen bacteria for microbial biohydrogenation. Regarding n-3 fatty acids and the n-6/n-3 ratio, we found that the PA group had the highest values for n-3 and the best n-6/n-3 ratio relative to the MS group, as well as high linolenic fatty acid content compared to LCPUFA n-3. The PH group had an intermediate n-3 fatty acid content and n-6/n-3 ratio, while LCPUFA n-3 showed a similar value compared to the PA group.

A decrease in the n-6/n-3 PUFA ratio as well as an increase in the PUFA/SFA was described by Leheska et al [10] for beef with the inclusion of grass in the diet. In our data, the two ratios considered for PA meat were in line with the recommendations from the World Health Organization (>0.45 for PUFA/SFA ratio and <5 for n-6/n-3). In addition, the beneficial effect linked to the presence of grass in the diet produces lower adipose accumulation, which results in significantly lower values of myristic acid and palmitic acid, thus significantly lowering the atherogenic and TI compared to MS, confirming the results of Giordano et al [2] regarding the beneficial effects of buffalo meat in limiting the onset of cardiovascular disease [3]. Additionally, other studies [13,26] found lower values for these fatty acids in grass-fed and meadow hay-fed animals. For MUFA fatty acids, only palmitoleic (C16:1) and octadecenoic acid (C18:1 cis-11) showed significant differences, having the lowest incidence for meat of the MS group, as referred to by Giuffrida-Mendoza et al [3]. The incidence of MUFA declines in muscle as fat deposition increases. MUFA fatty acids are positioned under intermediate conditions for human health considering their protective effect against lipid oxidation by PUFAs. The n-6 fatty acids that represent substrates for pro-inflammatory eicosanoids did not show significant differences between the experimental groups except for arachidonic acid (C20:4 n-6), which was abundant in animals fed corn silage [10]. These changes in fatty acid composition (particularly for LCPUFA n-3 and CLA) linked to PA feeding of buffalo were favourable regarding current human dietary guidelines [10].

The PCA confirms in the results obtained with the single variables, in particular from the analysis of the scores, the distance of MS with respect to the other two groups appears clear, and the overlap in part shows the beneficial effects of grazing and diet on nutritional values, while the MS group stands out in a positive manner for its light colour.

The PUFA n-3, CLA cis-9 trans-11, and n-6/n-3 values suggest that buffalo meat breeding on PA or good meadow hay is potentially more beneficial for nutrition or human health. These two feeding systems could respond to many consumer needs, though they did not present excellent production performance.

## Figures and Tables

**Table 1:** Chemical composition of feedstuffs

Item	MS	PH	PA	Maize grain	Protein concentrate
Dry matter (%)	34.5	86.9	36.6	88.1	87
Crude protein (%)	2.7	11.0	3.5	9.0	38
Ether extract (%)	1.1	1.6	1.1	3.5	3.5
Ash (%)	1.6	5.7	2.9	1.6	9.5
N-free extract (%)	21.6	44.1	17.6	71.4	25
Crude fibre (%)	6.5	23.4	12.5	2.6	12
NDF (%)	15.7	51.5	22.9	8.9	20.4
ADF (%)	9.0	28.8	14.1	3.2	9.5
C16:0	17.1	24.4	19.3	11.5	14.1
C18:0	3.5	5.8	4.2	1.8	3.8
C18:1cis-9	25.3	20.6	11.4	25.7	24.3
C18:2 n-6	46.2	28.3	22.5	55.9	49.6
C18:3 n-3	4.7	18.2	39.4	2.3	5.8

MS, maize silage; PH, polyphite meadow hay; PA, pastures; NDF, neutral detergent fiber; ADF, acid detergent fiber.

**Table 2:** Slaughter and carcass performances

Items	MS	PH	PA	RMSE	p-value
Live weight (kg)	256.6	256.0	255.9	16.63	0.995
Slaughter age (d)	331^b^	341^b^	384^a^	11.70	<0.001
GEC (kg)	21.6^c^	39.6^b^	43.0^a^	5.20	<0.001
ADG (kg/d)	0.65^a^	0.63^a^	0.58^b^	0.05	0.015
NLW (kg)	235.0^a^	216.5^b^	212.9^b^	16.30	0.011
Carcass weight (kg)	133.6^a^	118.7^b^	105.2^b^	10.73	<0.001
Dressing %	56.9^a^	54.7^ab^	49.5^b^	2.32	<0.001
Conformation point1)	6.33^a^	6.35^a^	5.26^b^	0.47	<0.001
Fat point1)	5.73^a^	3.54^b^	3.08^b^	0.74	<0.001
Meat (%)2)	58.46^b^	62.09^a^	60.61^a^	1.97	<0.001
Bone (%)2)	23.04^b^	24.37^b^	26.44^a^	1.51	0.012
Total fat (%)2)	14.45^a^	9.25^b^	8.60^b^	0.91	<0.001

MS, maize silage; PH, polyphite meadow hay; PA, pastures; RMSE, root mean standard error; GEC, gastro enteric contents; ADG, average daily gain; NLW, net live weight; Total fat, subcutaneous and intermuscular fat.

1) SEUROP classification.

2) Percentage on entire carcass (the complement to 100 is given by the other tissues).

a–c Means within a row without a common superscript letter differ (p<0.05).

**Table 3:** Comparison of anatomical regions

Parameters (%)	MS	PH	PA	RSME	p-value
Distal pelvic limb	7.19^b^	7.96^a^	8.24^a^	0.43	<0.001
Proximal pelvic limb	27.66	27.81	27.53	0.75	0.692
Loin	6.63	6.61	6.53	0.38	0.806
Abdominal region	4.68^a^	4.03^b^	3.72^b^	0.28	<0.001
Plate ribs 7–13	7.15	6.95	6.75	0.48	0.089
Brisket 7–13 rib	6.01	5.82	5.96	0.66	0.701
Flat ribs 1–6	9.21	8.72	8.91	0.55	0.147
Brisket 1–6	6.39	6.46	6.61	0.36	0.375
Shoulder	12.45	12.62	12.74	0.61	0.586
Neck	8.51	8.62	8.69	0.52	0.731
Fore shin	4.12^b^	4.40^a^	4.32^a^	0.18	0.006

MS, maize silage; PH, polyphite meadow hay; PA, pastures; RMSE, root mean standard error.

a,b Means within a row without a common superscript letter differ (p<0.05).

**Table 4:** Physical and chemical parameters of longissimus thoracis muscle

Parameters	MS	PH	PA	RMSE	p-value
pH	5.54^c^	5.62^b^	5.73^a^	0.08	<0.001
WHC (%)	1.16	1.03	1.20	0.28	0.415
Cooking loss (%)	30.55^a^	27.41^b^	28.88^ab^	1.65	<0.001
L*	46.81^a^	42.46^b^	41.10^b^	1.80	<0.001
a*	18.10^b^	19.65^a^	19.92^a^	1.16	0.003
b*	14.53^a^	12.60^b^	10.93^b^	1.12	<0.001
WBS raw (N)	50.73^b^	58.81^a^	60.20^a^	6.79	0.013
WBS cooked (N)	47.197	44.34	46.01	9.91	0.827
Dry matter (%)	22.66	23.41	23.06	0.97	0.125
Ash (%)	1.05	1.06	1.14	0.11	0.082
Ether extract (%)	1.18^a^	1.17^a^	0.67^b^	0.26	<0.001
Crude protein (%)	20.43	21.16	21.25	0.85	0.094
Total collagen (mg/g)	3.05^b^	3.80^a^	4.30^a^	0.59	0.004
Insoluble collagen (mg/g)	2.17	2.36	2.49	0.44	0.309
TBARS 7 d (mg MDA/kg)	0.16^a^	0.14^ab^	0.11^b^	0.04	0.033
TBARS 14 d (mg MDA/kg)	0.23^a^	0.15^b^	0.14^b^	0.04	<0.001

MS, maize silage; PH, polyphite meadow hay; PA, pastures; RMSE, root mean standard error; WHC, water-holding capacity; WBS, Warner–Bratzler shear force; TBARS, thiobarbituric acid reactive substances; MDA, malondialdehyde.

a–c Means within a row without a common superscript letter differ (p<0.05).

**Table 5:** Fatty acids composition of longissimus thoracis muscle (% of total fatty acid methyl ester)

%	MS	PH	PA	RMSE	p-value
C12:0	0.09	0.08	0.06	0.03	0.337
C14:0	1.15^a^	0.90^b^	0.76^b^	0.15	0.003
C15:0	0.21	0.23	0.23	0.04	0.213
C16:0	21.80^a^	19.97^b^	19.50^b^	1.04	0.050
C16:1	0.72^b^	1.13^a^	1.11^a^	0.25	0.002
C17:0	0.91	1.01	1.01	0.11	0.096
C17:1	0.43	0.42	0.46	0.07	0.486
C18:0	20.72	20.84	19.83	2.10	0.366
C18:1 trans-9	0.08	0.11	0.12	0.06	0.214
C18:1 cis-9	33.42	34.01	34.76	2.56	0.892
C18:1 cis-11	0.50^b^	0.85^a^	0.97^a^	0.19	<0.001
C18:2 n-6	13.18	12.84	12.99	2.57	0.983
CLA cis-9trans-11	0.22^c^	0.29^b^	0.35^a^	0.05	<0.001
C18:3 n-3	0.77^c^	1.07^b^	1.32^a^	0.31	<0.011
C18:3 n-6	0.20	0.19	0.18	0.07	0.827
C20:0	0.13	0.17	0.16	0.04	0.064
C20:3 n-6	1.02	1.08	1.21	0.35	0.067
C20:4 n-6	3.17	2.76	2.68	0.90	0.390
C20:5 n-3	0.22^c^	0.18^b^	0.57^a^	0.12	0.004
C22:4 n-6	0.52	0.59	0.55	0.13	0.609
C22:5 n-3	0.49^b^	0.85^a^	1.02^a^	0.26	0.006
C22:6 n-3	0.05^b^	0.13^a^	0.16^a^	0.09	0.021
SFA	45.01	43.20	41.55	2.23	0.064
MUFA	35.15	36.52	37.42	3.74	0.636
PUFA	18.84	20.28	21.03	3.86	0.761
PUFA/SFA	0.42	0.47	0.51	0.11	0.479
PUFA n-6	19.09	17.46	17.61	3.47	0.517
PUFA n-3	1.53^c^	2.53^b^	3.07^a^	0.58	0.003
n-6/n-3	12.47^a^	6.90^b^	5.74^c^	1.37	<0.001
AI1)	0.48^a^	0.42^ab^	0.39^b^	0.04	0.003
TI2)	1.32^a^	1.20^ab^	1.09^b^	0.15	0.006

MS, maize silage; PH, polyphite meadow hay; PA, pastures; RMSE, root mean standard error; SFA, saturated fatty acids; MUFA, mono unsaturated fatty acid; PUFA, poly unsaturated fatty acid.

1) AI, atherogenic index = [C12:0+(4×C14:0)+C16:0]/[(∑PUFA)+ (∑MUFA)].

2) TI, thrombogenic index = [C14:0+C16:0+C18:0]/[(0.5×∑MUFA)+(0.5×n6) +(3×n3)+(n3/n6)].

a–c Means within a row without a common superscript letter differ (p<0.05).
